# Dual-Responsive Alginate Hydrogel Constructed by Sulfhdryl Dendrimer as an Intelligent System for Drug Delivery

**DOI:** 10.3390/molecules27010281

**Published:** 2022-01-03

**Authors:** Li Li, Dongyu Lei, Jiaojiao Zhang, Lu Xu, Jiashan Li, Lu Jin, Le Pan

**Affiliations:** 1 Chemical Engineering College, Xinjiang Agricultural University, Urumqi 830052, China; lizixi19950707@163.com (L.L.); koh9391421@163.com (J.Z.); lur281644038@163.com (L.X.); lijiashan3333@163.com (J.L.); 2 The Key Laboratory of Synthetic and Biological Colloids, Ministry of Education, Jiangnan University, Wuxi 214122, China; 3 Department of Physiology, Preclinical School, Xinjiang Medical University, Urumqi 830011, China; leidongyu0109@163.com

**Keywords:** dendrimer modified hydrogel, GSH- and pH-responsive, anti-tumor activity

## Abstract

Intelligent stimulus-triggered release and high drug-loading capacity are crucial requirements for drug delivery systems in cancer treatment. Based on the excessive intracellular GSH expression and pH conditions in tumor cells, a novel glutathione (GSH) and pH dual-responsive hydrogel was designed and synthesized by conjugates of glutamic acid-cysteine dendrimer with alginate (**Glu-Cys-SA**) through click reaction, and then cross-linked with polyethylene glycol (PEG) through hydrogen bonds to form a 3D-net structure. The hydrogel, self-assembled by the inner disulfide bonds of the dendrimer, is designed to respond to the GSH heterogeneity in tumors, with a remarkably high drug loading capacity. The Dox-loaded **Glu-Cys-SA** hydrogel showed controlled drug release behavior, significantly with a release rate of over 76% in response to GSH. The cytotoxicity investigation indicated that the prepared DOX-loaded hydrogel exhibited comparable anti-tumor activity against HepG-2 cells with positive control. These biocompatible hydrogels are expected to be well-designed GSH and pH dual-sensitive conjugates or polymers for efficient anticancer drug delivery.

## 1. Introduction

Currently, wide ranges of anticancer drugs receive accelerated approval every year. However, the adverse side effects due to the poverty of pharmacological targets remain serious problems that are difficult to overcome [1,2]. Besides this, the drawbacks include low water solubility, drug stability, and high efflux from cells, which constrain the therapeutic effects of drugs in tumor treatment [3]. Therefore, how to deliver drugs to the target site (tissues or cells) and release therapeutic concentrations efficiently and safely are the main challenges for innovations to chemotherapeutic agents.

The discovery and design of stimulus-sensitive agents according to the differences of pH and redox potential between normal cells and tumor cells has been extensively explored in recent years [4,5,6,7]. Tumor cells are found to overproduce intracellular glutathione (GSH) or reactive oxygen species (ROS) to maintain a strongly reducing environment or increased oxidative stress. It is interesting that overproduced GSH and ROS could exist in different tumors, in different regions in the same tumor, or at different stages during tumor growth. Based on this, GSH/ROS-responsive targeted drug release has been thought to be an attractive method for the discovery of anti-tumor agents. Thiolysis of the disulfide bond by GSH or the oxidation of thioether to hydrophilic sulfoxide have triggered the design and syntheses of intelligent biopolymer systems [8].

Hydrogels have been developed into an appealing material for biomedical applications in the fields of the medicine and pharmaceutical industries [9,10]. Alginate is a highly polar linear biopolymer with monomeric units of β-dmannuronic acid (M) and α-L-guluronic acid (G), which are connected by β (1–4) glycosidic bonds and α (1–4) bonds (GG and GM). The carboxylate and hydroxyl group units benefit the functionalization of the polymeric by conjugating with different responsive groups [11]. Recently, in situ-forming multi-responsive hydrogels had burst forth as being of crucial importance in targeted drug delivery systems. The dynamic properties of non-covalent bonds with breaking and forming equilibriums under certain conditions have provoked intrigue as to their applications to construct biofunctional injectable hydrogels. The disulfide bond is widely used to form typical intelligent gelation through self-repairing, shape-recovery, and dynamic recombination [12]. Inspired by these findings, a glutamic acid–cysteine dendrimer (**Glu-Cys** dendrimer) was designed and synthesized, and a self-healing hydrogel based on the disulfide bonds derived from the **Glu-Cys** dendrimer conjugating with alginate (**Glu-Cys-SA**) was proposed to be designed and synthesized by click chemistry. By this, the loading capacity could be improved by the structure of dendrimer, and the target release at the tumor site was achieved by GSH-responsive dynamic cross-linking networks.

## 2. Materials and Methods

### 2.1. Materials and Agents

L-Cysteine, 2-(1H-Benzotriazole-1-yl)-1,1,3,3-tetramethyluronium hexafluoro-phosphate (HBTU), *N,N*-Diisopropylethylamine (DIPEA), *N*-(3-Dimethylamino-propyl)-*N′*-ethylcarbodiimide hydrochloride (EDC), *N*-Hydroxysuccinimide (NHS), and L-Glutathione (GSH) were purchased from MACKLIN (Shanghai, China). The 1-Hydroxybenzo-trizole (HOBt) were from Damas Beta (Shanghai, China). H-Glu(OBzl)-Obzl, Boc-L-Glutamic acid, and Doxorubicin hydrochloride (DOX) were from Aladdin (Shanghai, China). Sodium alginate (SA) was from Tianjin Beilian Fine Chemicals Development Co. Ltd. (Tianjin, China) The other starting chemicals were purchased from commercial vendors, all of which were of AR grade and used without purification. Reactions were monitored by TLC (Silica Gel 60 F254). The prepared compounds were purified with column chromatography using silica gel (300–400 mesh). ^1^H-NMR (400 MHz) and ^13^C-NMR (100 MHz) spectra were recorded with a Bruker AM-400BB spectrometer (Bruker, Karlsruhe, Germany) with TMS as the internal standard. Chemical shift values (δ) were listed in ppm and the coupling constant values (*J*) are in Hertz. The morphology of the freeze-dried hydrogel was observed by scanning electron microscope. The release properties were investigated by UV spectrophotometer.

### 2.2. Synthesis of Glu-Cys Dendrimer

The **Glu-Cys** dendrimer was constructed by the condensation reaction between the cysteine and L-glutamic acid [13].

**Scheme 1:**
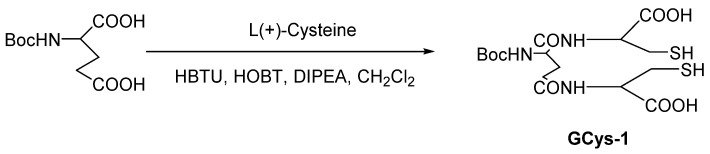
Synthesis of **Gcys-1**.

**Synthesis of Gcys-1.** BOC-L-glutamic (5 mmol) was dissolved with CH_2_Cl_2_ in 250 mL round bottom flask. HBTU (15 mmol) and HOBT (15 mmol) were added with stirring in ice bath under the protection of vacuum nitrogen. After 30 min, DIPEA (20 mmol) was added with stirring for 20 min followed by drop addition of L-Cysteine (15 mmol). After 30 min, the reaction system was raised to room temperature. After the reaction, the above reaction solution was alternately washed with saturated sodium bicarbonate solution and 5% sodium bisulphate, three times. The solvent was removed by rotary evaporation and purified by column chromatography to obtain **Gcys-1** (Scheme 1).

**Scheme 2:**
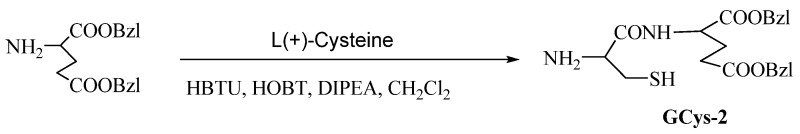
Synthesis of **Gcys-2**.

**Synthesis of Gcys-2.Gcys-2** was obtained as white solid with a method similar to that of **Gcys-1** by the reaction between H-Glu(Obzl)-Obzl·HCl and L-Cysteine (Scheme 2).

**Scheme 3:**
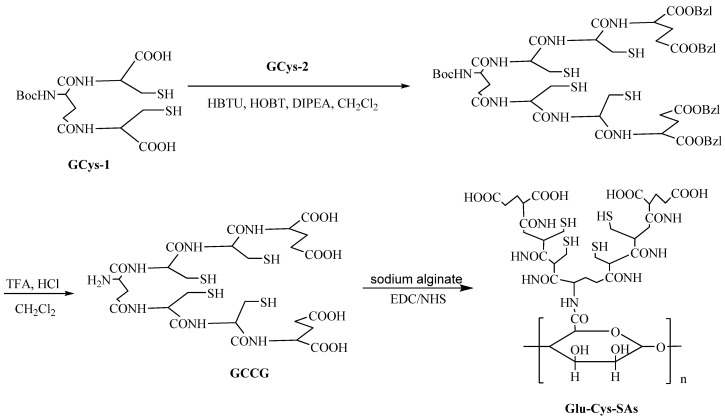
Synthesis of **GCCG**.

**Synthesis ofGlu-Cys dendrimer (GCCG).Glu-Cys** dendrimer (**GCCG**) was synthesized by the condensation reaction between the carboxyl group of **Gcys-1** and the primary amine group of **Gcys-2**. **Gcys-1** (10 mmol) was dissolved in CH_2_Cl_2_ and its carboxyl groups were activated by adding HBTU (20 mmol) and HOBT (40 mmol). DIPEA (40 mmol) was drop-added with stirring followed by adding **Gcys-2** (10 mmol). After the reaction, the above reaction solution was alternately washed with saturated sodium bicarbonate solution and 5% sodium bisulphate, three times. The white solid was obtained after purification with column chromatography. The yields were further deprotection in the presence of TFA and HCl at 25 °C under the protection of *N*_2_. Finally, **GCCG** was obtained as white solid [14,15,16] (Scheme 3).

### 2.3. Preparation of Glu-Cys-SA

**Glu-Cys-SA** hydrogel was prepared with the click reaction between the amine group of **Glu-Cys** dendrimer and carboxyl group of SA (Scheme 3). Briefly, the sodium alginate (SA) (150 mg) was dissolved in 4 mL deionized water, and then DMSO dissolved arbodiimide (EDC) and N-Hydroxysuccinimide (NHS) (SA: EDC: NHS = 1:1.1:1.1) were added with stirring to activate the carboxyl groups. The prepared **Glu-Cys** dendrimer was dissolved with DMSO and dropped in to the activated-SA solution with stirring at 10 °C in the dark (SA: **Glu-Cys** = 1:1.5) [17]. After the reaction, the pH of solution was adjusted to 6 with HCl, followed by precipitation with anhydrous ethanol. Finally, the solid powder was prepared and freeze-dried to obtain loose powder. Series of **Glu-Cys-SA** polymers with different sulfhydryl group ratios were synthesized by adjusting the amount of **Glu-Cys**. The poly (ethylene glycol) (PEG) was introduced to form hydrogen-bond interaction between the thiol groups and the hydroxyl group of PEG.

### 2.4. FT-IR Spectroscopy

**Glu-Cys** dendrimers and **Glu-Cys-SAs** were dried, mixed with KBr, and ground into powders. Fourier-transform infrared spectrometer was applied to characterize the prepared **Glu-Cys-SA** and DOX-loaded samples with SA as blank in the wavelength range of 4000–500 cm^−1^ [18].

### 2.5. Capillary Flow Test and Tube Inversion

To find the best gel concentration, capillary flow test is used. **Glu-Cys-SAs** was dissolved in 0.5 mL deionized water in a 2.5 mL sample bottle to prepare a solution with a concentration of 1–5%. At room temperature, the capillary with an inner diameter of 0.3 mm was immersed vertically into the dispersion and measured every 5 min until the height of the capillary remained unchanged. Gelation threshold is the point at which no dispersion rises to the capillary, indicating the critical gelation concentration. Besides, tube inversion method was applied to determine the visual gel time [19].

### 2.6. Size and Morphology of Hydrogel

The morphology of the hydrogels was studied by scanning electronic microscopy (SEM, HITACHI S-800, Hitachi, Japan). The hydrogel samples were prepared in molds at room temperature and lyophilized to sponges for SEM observation. The obtained sponges were cross-sectioned and sputter-coated with a thin layer of gold. Pore size of hydrogels were then measured [20].

### 2.7. Self-Healing Investigation

The capacity of self-healing of the hydrogel was explored by punching tests. The swollen hydrogels were put into the sample molds. A disc was made in the center of the gel with a 0.7 cm punch and put back at room temperature. After taking pictures, the self-healing properties were investigated by observing whether the gel became a whole piece without cracks after a period of time until fully repaired [21].

### 2.8. GSH Responsiveness Measurement

GSH were used as the reductant to investigate the redox responsiveness. The **Glu-Cys-SAs** hydrogel was immersed in two different buffers, one of which was a blank buffer solution and the other was the buffer solution containing 10 mM glutathione. At set intervals, the gel was removed, dried, weighed, and put it back into the buffer solution until reaching swelling equilibrium [12]. The corresponding properties were examined.

### 2.9. pH Responsive Transition of Hydrogels

The hydrogels were prepared in molds and then placed into different media (pH 9, pH 7.4 and pH 5.5) to mimic the intracellular pH entracellul. The swelling or deswelling properties in response to pH were observed at specific time intervals and size changes were recorded. The swelling or deswelling ratios (SR) were measured with the equation: SR = (Wv − Ws)/Ws (Wv: at specific time intervals, the hydrogels were taken out from the tubes and gently blotted with filter paper to remove the excess of water lying on the surfaces, and weighed; Ws: hydrogels’ initial weight) [12,22].

### 2.10. Drug Release

For drug release studies, xerogel was put in a flask vial and 0.5 mL of 10 mg/mL doxorubicin hydrochloride solution was added with a constant heat at 37 °C. After loading for about 24 h, the free doxorubicin hydrochloride on the surface was washed with deionized water, followed by immersing in 3 mL PBS buffer solution with different treating (pH 5.5, pH 7.4, GSH + pH 5.5, and GSH + pH 7.4) for drug release test. At intervals, 2 mL samples were withdrawn from the solution to measure the concentration of doxorubicin hydrochloride with an ultraviolet absorption spectrum, and the removed volume from the vial was replaced with PBS to keep the condition stable. The standard curve was prepared with doxorubicin hydrochloride. Finally, the release curve was drawn [18,23,24,25].

### 2.11. In Vitro Cytotoxicity Investigation

The cytotoxicity against HepG2 cells and L929 cells of the prepared hydrogel was evaluated with MTT assay. The cells were seeded in 96-well plates at a density of 10^4^ cells/well. Hydrogels of different concentrations (0.5, 1 and 5 mg/mL) were added into each well. After being incubated for 24 h, 48 h, and 72 h at 37 °C, 10 μL/well of MTT (5 mg/mL) was added and the cells were incubated for 4 h at 37 °C. Absorbance at 490 nm was measured using SPECTRAmax microplate spectrum. Cell viability was expressed relative to control without any sample addition. Free doxorubicin was applied as positive control.

## 3. Results and Discussion

### 3.1. The Synthesis of Glu-Cys Dendrimer Fabrication and Glu-Cys-SA Hydrogel

**Figure 1:**
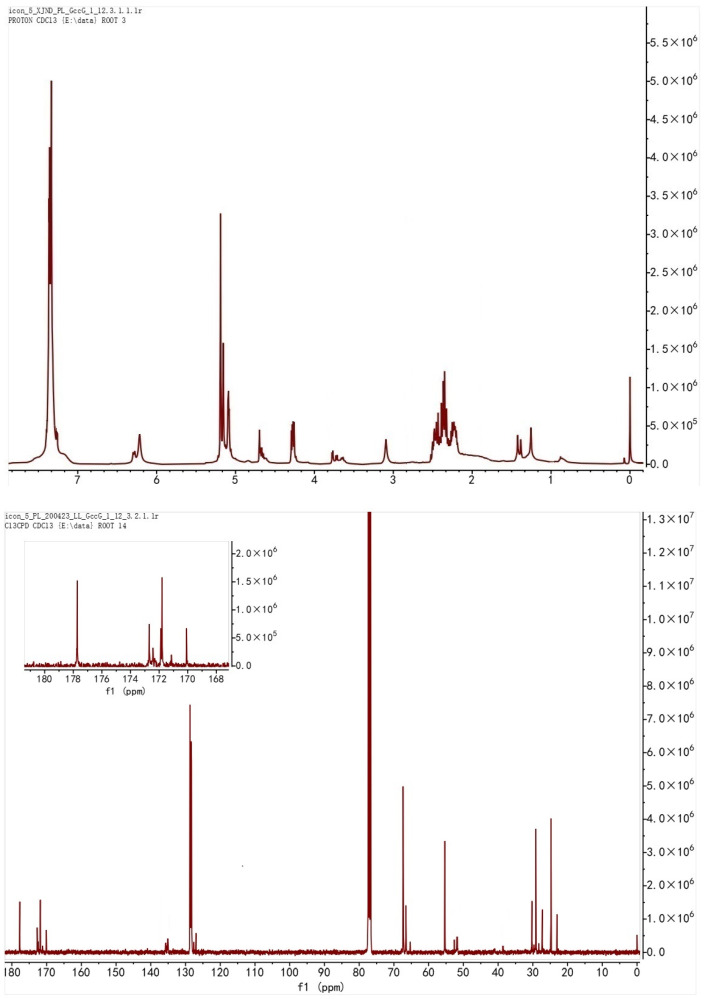
The 1 H NMR and 13 C NMR of dendrimer **GCCG**.

The compounds **Gcys-1**, **Gcys-2,** and **Glu-Cys** dendrimer (**GCCG**) were successfully synthesized with high yields. The glutamic acid was chemically conjugated with L-Cysteine by click reaction between the primary amine group of L-Cysteine and the carboxyl group of glutamic acid [26,27,28]. The chemical structures of intermediate product and **GCCG** were confirmed by ^1^H NMR and ^13^C NMR (Figure 1). The introduction of PEG significantly increased the strength of hydrogel by forming hydrogen-bond interactions between the thiol groups and the hydroxyl group of PEG. With this method, the dendrimer-modified hydrogel could be efficiently prepared.

### 3.2. FT-IR Characterization

**Figure 2:**
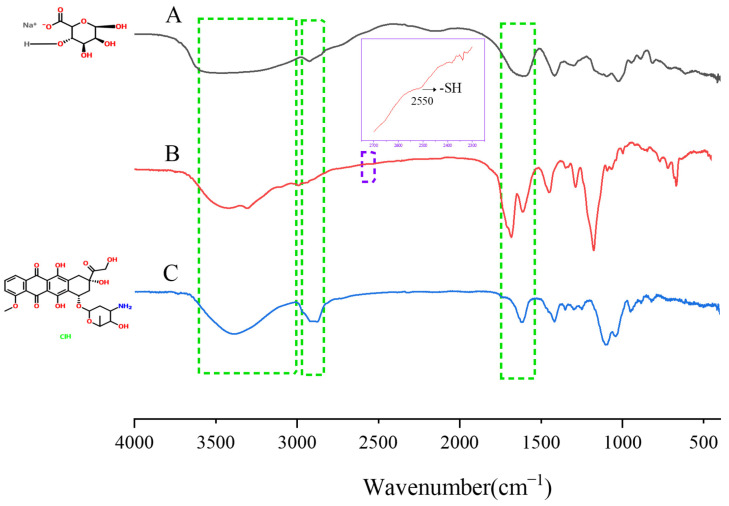
Infrared spectra of samples: A, sodium alginate; B, the prepared dendrimer-modified hydrogel (**Glu-Cys-SAs**); C, dox-loaded **Glu-Cys-SAs**.

FT-IR spectra were applied to characterize the structures of DOX-loaded hydrogels with the assignment of diagnostic peaks (Figure 2). The broad bands in the 3600–3000 cm^−1^ region is correlated to O-H and N-H stretching vibrations. C-H telescopic vibration absorption peaks were found at 2923 cm^−1^ due to the tension in the six-membered ring in the sugar unit of sodium alginate. S-H vibration absorption peaks occur at 2550 cm^−1^, though it is weak due to the low relative content. The peak at 1633 cm^−1^ is correlated to the residual N-acetyl group from alginate (C=O stretching of amide). The results confirmed the successful forming of an amide bond and the loading of doxorubicin.

### 3.3. Dynamic Properties of Hydrogels

**Figure 3:**
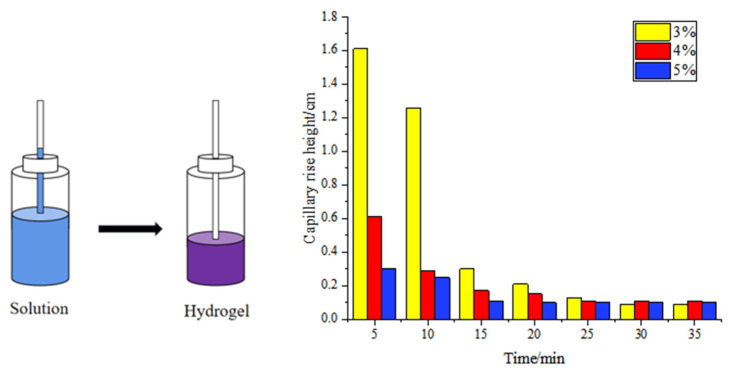
Schematic diagram of the capillary measurement gel formation and Sol–gel transition properties characterized by capillary measurement.

**Figure 4:**
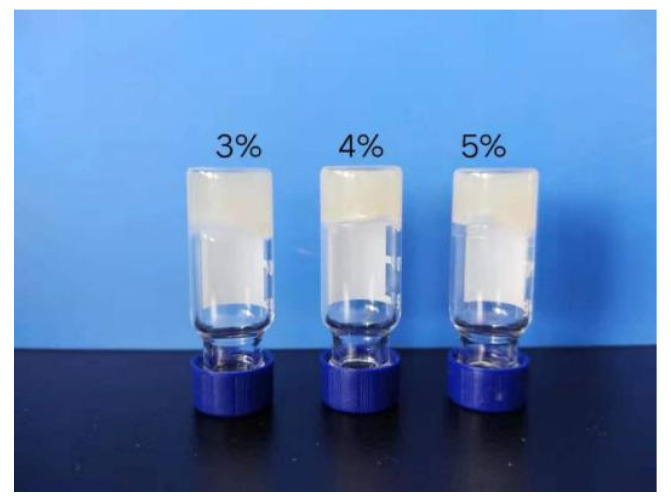
Tube inversion test at different gel concentrations (3%, 4% and 5%).

The capillary method and the tube inversion method were applied to determine the gelation threshold and characterize the sol–gel transition kinetics. The capillary measurement is based on the capillary phenomenon. The rise height of the sample in the capillary tube depends on the particle size and the topological structure of the liquid. For our **Glu-Cys-SAs** system, the kinetic properties can be reflected by the rise height in the capillary. It is indicated that the gel time decreased sharply with the increase of concentration (Figure 3). The tube inversion revealed that the prepared gels could facilitate rapid fixation at the intracellular of 3–5 mg/mL within a few minutes. This was in accordance with the result of capillary test (Figure 4).

### 3.4. Size and Morphology of Hydrogel

**Figure 5:**
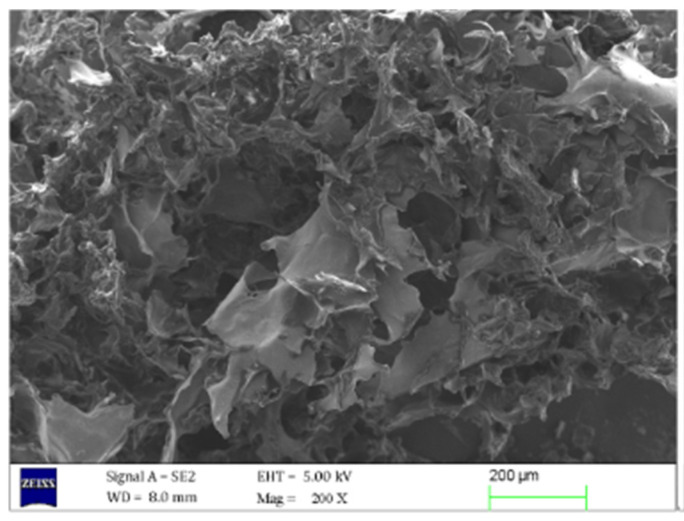
SEM micrographs of **Glu-Cys-SAs** gel.

The pore structure and appropriate microscopic morphologies contribute highly to the properties of hydrogels, including load capacity, nutrient release, and mechanical properties. Compared to original mass, the water retention was up to 25-times higher after reaching swelling equilibrium at 24 h, indicating that the highly cross-linked structure contributed to the swelling capacity of hydrogels. A denser pore structure was found, as observed in SEM (Figure 5).

### 3.5. Self-Healing Properties

**Figure 6:**
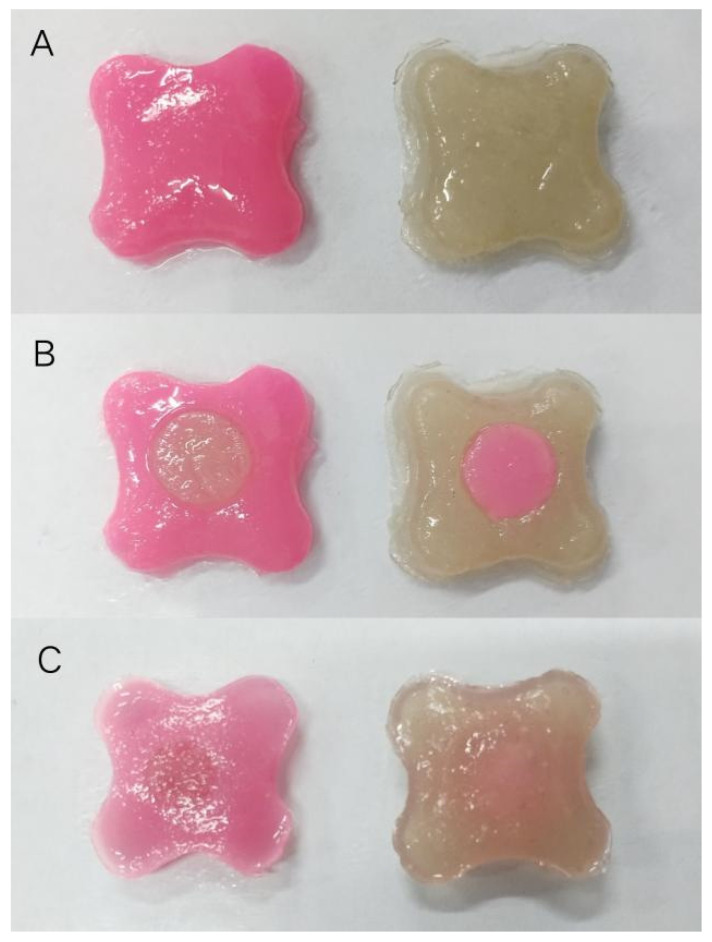
Self-healing properties of hydrogels: (**A**) the prepared hydrogel gel; (**B**) disc placed back into hole for self-repairing at room temperature; (**C**) fully repaired gel.

**Figure 7:**
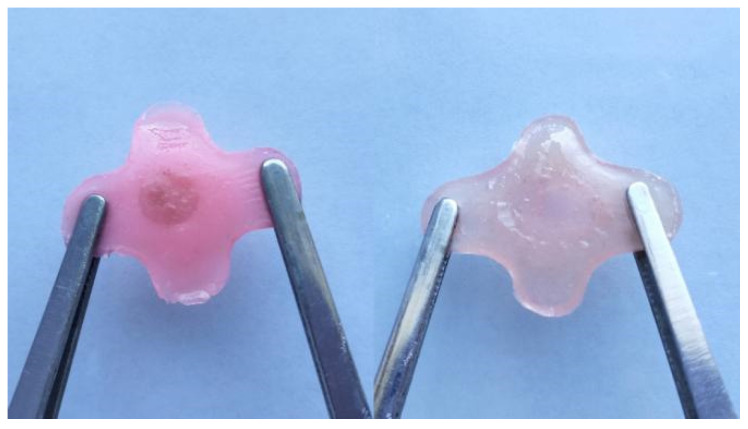
Stretch test.

The dual-responsive hydrogel with self-healing properties was crucial for drug loading and delivery. A macroscopical experiment was employed to investigate the self-healing performance. As the results show, the punched disc could stick back to the hydrogel and the crack finally entirely disappeared, which also could afford some tension to a certain extent (Figure 6 and Figure 7). It was interesting that the oxidation condition could enhance the self-healing, which was in accordance with the result of the redox-responsive experiment. According to the chemical structure of the **Glu-Cys-SAs**, it could be concluded that the rapid self-healing was mainly caused by electrostatic interaction and disulfide bonds due to the high-content carboxyl group and the sulfhydryl group in the system.

### 3.6. GSH- and pH-Responsive Reversible Transition

**Figure 8:**
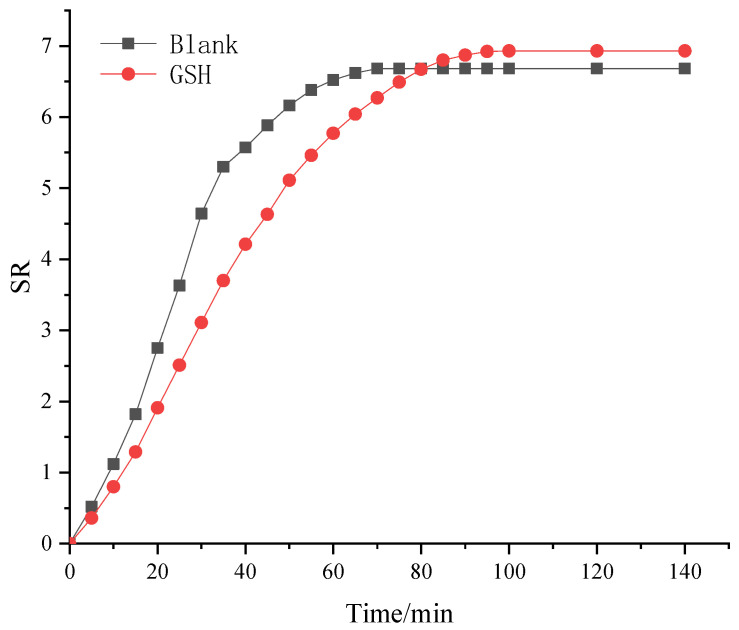
Gel swelling equilibrium in GSH response.

Because the prepared hydrogels aimed to be intracellular redox-responsive according to the reductively degradable disulfide bonds, the stimulus responsive behavior was investigated to understand its properties. It was found that hydrogel was difficult to construct when GSH was added at the preliminary stage. The figure (Figure 8) shows the swelling curve of the gel in the buffer solution containing 10 mM GSH and the blank buffer solution. It indicated that the gel reached equilibrium in the buffer solution, which is faster than that in the buffer solution containing 10 mM GSH. Meanwhile, the sticky edge was observed on the hydrogel immersed in GSH containing the buffer solution. Therefore, GSH induced disulfide cleavage and resulted in the break of network structures.

**Figure 9:**
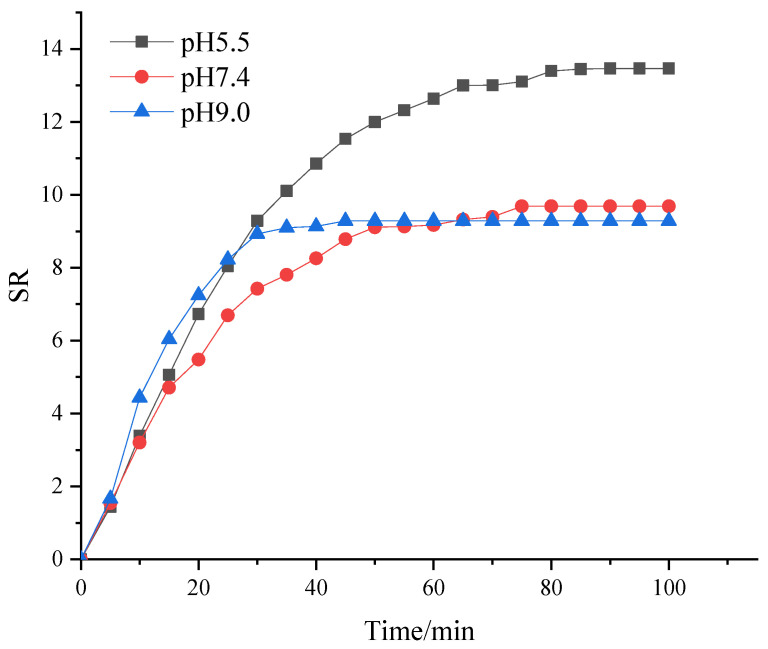
pH responsive properties of gel swelling equilibrium at pH 5.5, pH 7.4, and pH 9.0.

Good pH-responsive properties of the prepared hydrogels were observed according to pH gradient (Figure 9). It was found that it took only 30 h to reach swelling equilibrium at pH 9, whereas it took over 60 h at pH 5.4. With the pH value of the solution increased from 5.5 and 7.4 to 9.0, it took more time to reach swelling equilibrium. The main reason was that, in acid condition, the carboxyl groups could be formed, and the molecular chain gradually changed from a winding to a fully extended state due to intermolecular repulsion.

### 3.7. GSH- and pH-Dependent In Vitro Release Test of DOX-Loaded Glu-Cys-SA Hydrogel

The prepared hydrogels were found to exhibit excellent GSH- and pH-responsive drug release properties. The release of **Glu-Cys-SA** carrier gum was treated with the following two pH conditions: physiological conditions (pH 7.4) and an acidic condition (pH 5.5). It was found that the accumulated release rate reached 35% at pH 7.4, whereas a significant release speed was observed under acidic conditions (pH 5.5) up to 56%. The result indicated that the drug release is highly pH sensitive.

**Figure 10:**
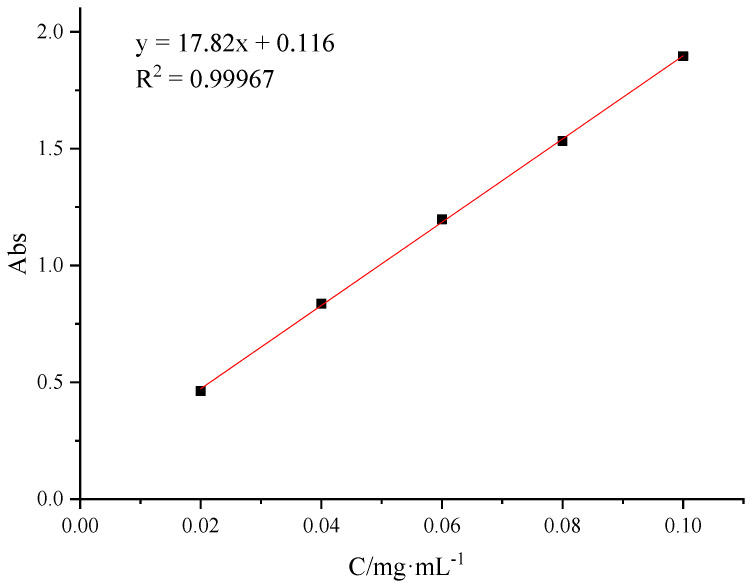
Standard curve of amamubicin hydrochloride.

**Figure 11:**
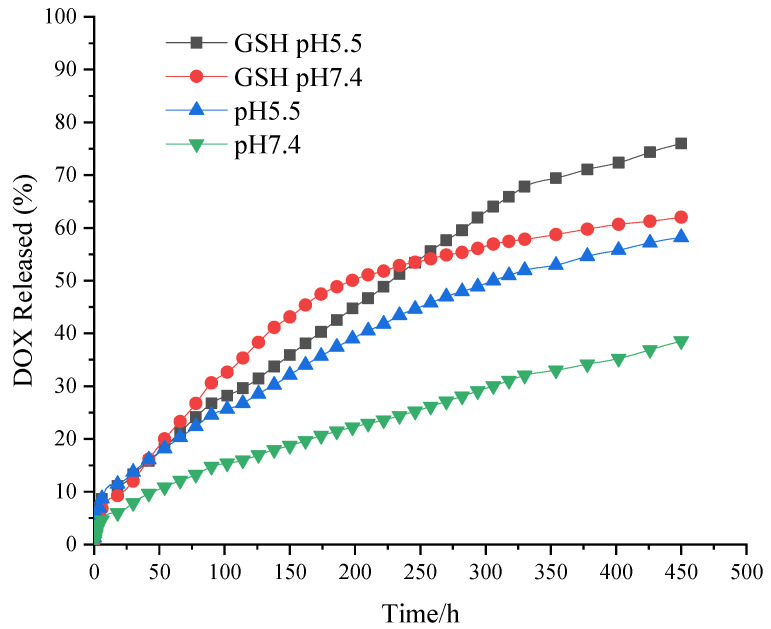
The Dox-loaded hydrogel was investigated for controlled release in PBS solutions having GSH + pH 5.5, GSH + pH 7.4, pH 5.5 and pH 7.4.

Meanwhile, it is more important to study the reversible sol–gel transition behaviors in response to GSH condition, which is much higher for intracellular conditions in cancer cells than in the bloodstream. The cell’s exterior stable disulfide bonds could be cleaved in the reductive intracellular environment due to excessive GSH expression. The prepared hydrogels exhibited an excellent dual-responsive property, including GSH sensitivity. The drug release of the **Glu-Cys-SA** carrier in 10 mM GSH at pH 7.4 (GSH + pH 7.4) and 10 mM GSH at pH 5.5 (GSH + pH 5.5) were investigated here. The release curve indicated that the accumulated release was about 62% in 10 mM GSH at pH 7.4, whereas the accumulated release rose to about 76% in 10 mM GSH at pH 5.5 (Figure 10 and Figure 11). Therefore, it could be found that the cumulative release rate of GSH participation increased significantly under the same pH conditions. It is indicated that, in the GSH and acidic condition, the disulfide bond was gradually cleaved, which resulted in the deconstruction of the network, and doxorubicin wrapped inside the gel was gradually released.

### 3.8. Evaluation of Cytotoxic Effect of Hydrogels

**Figure 12:**
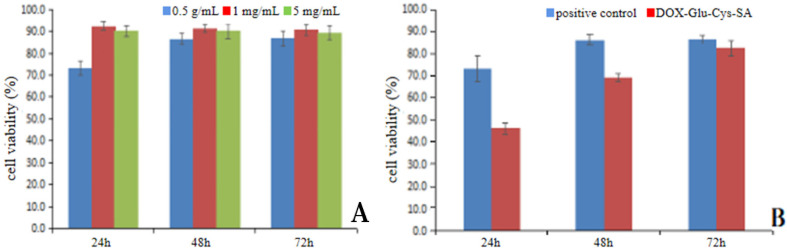
Cytotoxic effect of hydrogels: (**A**) the cytotoxicity of the prepared hydrogel against L929 cell; (**B**) the in vitro anti-tumor cytotoxicity of DOX-loaded hydrogel against Hep G2 cells.

In vitro anticancer effects of prepared hydrogels with/without Dox were investigated by the MTT assay against HepG-2 cells and the cytotoxicity of samples were investigated against L929 cells. It is found that the hydrogel without the doxorubicin showed no significant change on the L929 cell viability at concentrations up to 5 mg/mL, which indicated that the prepared hydrogel was good for biocompatibility, whereas a time-dependent and concentration-dependent cytotoxic effect were found in the treatment of DOX-loaded hydrogels. It is also interesting to note that the cytotoxicity of DOX-loaded hydrogel is weaker than that of positive control (free doxorubicin) at the earlier stage (24 h), whereas it is comparable after 72 h treatment. It is indicated that DOX-loaded **Glu-Cys-SA** could be safe and efficient for drug release (Figure 12).

## 4. Conclusions

In this work, smart dual-responsive cross-linked hydrogels were prepared from dendrimer-modified alginate (**Glu-Cys-SA**). FT-IR and NMR studies confirmed the composition and chemical modification of hydrogels. Doxorubicin was successfully incorporated into hydrogels and released in response to the pH and GSH condition. The pH and GSH environment significantly affected the mechanical properties and drug-release potential of the hydrogels. The total release of the drug was up to 76% in response to pH and GSH. The cytotoxicity study shows that the hydrogel showed no cytoxicity against normal cells, whereas it exhibited significant in vitro anti-tumor effects with doxorubicin-loading. Thus, the prepared self-healing hydrogels with GSH and pH dual-responsivity are potentially innovative for targeted delivery of drugs.

## Data Availability

Not applicable.
